# Can Cryopreservation in Assisted Reproductive Technology (ART) Induce Epigenetic Changes to Gametes and Embryos?

**DOI:** 10.3390/jcm12134444

**Published:** 2023-07-02

**Authors:** Romualdo Sciorio, Claudio Manna, Patricia Fauque, Paolo Rinaudo

**Affiliations:** 1 Edinburgh Assisted Conception Programme, Royal Infirmary of Edinburgh, Edinburgh EH16 4SA, UK; 2 Biofertility IVF and Infertility Center, 00198 Rome, Italy; 3 Université Bourgogne Franche-Comté—Equipe Génétique des Anomalies du Development (GAD) INSERM UMR1231, F-21000 Dijon, France; 4 CHU Dijon Bourgogne, Laboratoire de Biologie de la Reproduction—CECOS, F-21000 Dijon, France; 5 Department of Obstetrics, Gynecology, and Reproductive Sciences, University of California, San Francisco, CA 92037, USA

**Keywords:** human in-vitro fertilization (IVF), assisted reproductive technology (ART), cryopreservation procedure, vitrification, epigenetics modifications, offspring health

## Abstract

Since the birth of Louise Brown in 1978, more than nine million children have been conceived using assisted reproductive technologies (ARTs). While the great majority of children are healthy, there are concerns about the potential epigenetic consequences of gametes and embryo manipulation. In fact, during the preimplantation period, major waves of epigenetic reprogramming occur. Epigenetic reprogramming is susceptible to environmental changes induced by ovarian stimulation, in-vitro fertilization, and embryo culture, as well as cryopreservation procedures. This review summarizes the evidence relating to oocytes and embryo cryopreservation and potential epigenetic regulation. Overall, it appears that the stress induced by vitrification, including osmotic shock, temperature and pH changes, and toxicity of cryoprotectants, might induce epigenetic and transcriptomic changes in oocytes and embryos. It is currently unclear if these changes will have potential consequences for the health of future offspring.

## 1. Introduction

Over the past forty years, ART has been steadily on the rise, allowing millions of infertile couples to conceive. Currently, it is estimated that over nine million children have been conceived using ART [1,2]. While the number of IVF cycles varies widely worldwide, approximately 5% of births are secondary to the use of ART in some European countries [2]. The main driver of IVF utilization is individuals being affected by infertility (approximately 15% of couples). However, there is a continuous rise in the number of individuals who freeze their eggs or embryos for future use [2,3,4,5,6,7]. For example, nearly 310,000 frozen embryo transfer (FET) cycles were performed in Europe in 2018 [2]. 

Current evidence indicates that ART is safe; however, an association between ART and an increased incidence of low birth weight, birth defects, altered growth, and metabolic disorders has been reported [8,9]. These findings might be secondary to epigenetic dysregulation of gametes and embryos [10,11,12]. Given the continuous rise in the number of cycles that involve oocytes and embryo cryopreservation, it is critical to understand whether cryopreservation is harmful to the future health of children. In this manuscript, we describe the impact that vitrification has on potential epigenetic modifications and consequences for future offspring health. 

## 2. Increased Use of Oocyte and Embryo Vitrification in ART Practice

The advancements in oocyte cryopreservation found a perfect application for fertility preservation for social reasons or in patients affected by cancer. Indeed, societal changes have resulted in the postponement of the age of the first pregnancy [1,2], at the time when diminished ovarian reserve significantly reduces the chance of success. Therefore, multiple patients aim to freeze their eggs for future use. In the UK, elective egg freezing is the fastest growing fertility treatment, with an increase of 10% per year [13]. In Spain, egg freezing cycles increased from 4% of total vitrification procedures to 22% in 10 years [14]. In the USA, fertility preservation cycles increased from 9607 in 2017 to 13,275 in 2018; similar trends have been observed in other countries [15,16]. 

Egg freezing is also used by young cancer patients, since treatment for malignancies might negatively affect future fertility [17,18]. According to the International Agency for Research on Cancer, in 2020, there were an estimated 19.3 million new cancer cases, with nearly 10 million cancer deaths. Female breast cancer has surpassed lung cancer as the most commonly diagnosed cancer, with more than two million new cases per year [17]. 

Another important application of oocyte cryopreservation is in egg donor programs [19]. Since the description by Trounson of the first successful pregnancy following oocyte donation in Australia [20], the number of oocyte donation cycles has doubled in the last decade. For example, in the USA, the number of cycles increased from 10,801 in 2000 to 24,300 in 2016 and 49,193 in 2017 [21]. In 2017, 17,099 donors underwent an average of 2.4 oocyte collections [22]. The need for finding a large number of egg donors has resulted in the creation of multiple oocyte banks. In particular, an oocyte bank performs the egg retrieval and cryopreservation of oocytes, which are later transported to the receiving clinic. Then, the imported oocytes, in the IVF laboratory of the recipient center, are warmed, fertilized with the ICSI technique using fresh or frozen sperm, cultured, and transferred to the recipient’s uterus or possibly biopsied for PGT procedure and frozen again [23,24,25,26].

**Figure 1:**
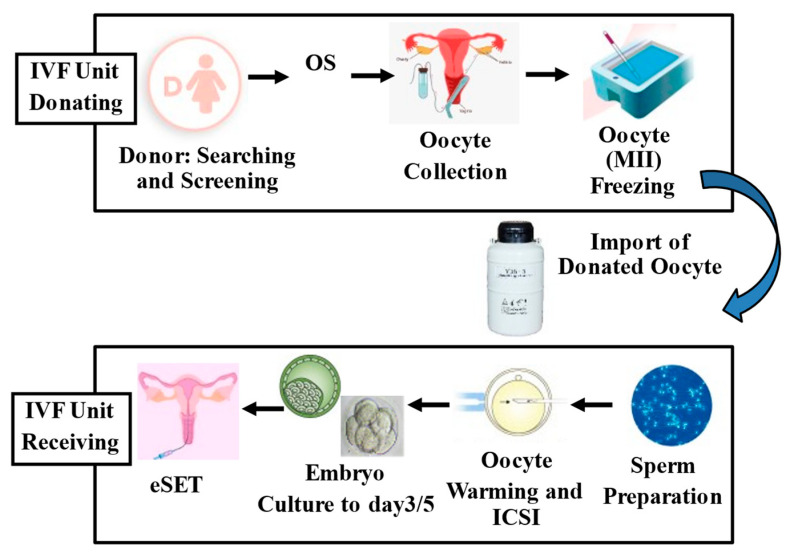
Description of the imported oocyte donation program from a foreign oocyte bank. eSET, elective single embryo transfer; ICSI, intracytoplasmic sperm injection; MII, metaphase II oocyte; OS, ovarian stimulation.

Several studies have analyzed the efficiency of oocyte vitrification. Importantly, egg donor vitrification provides high survival rates after warming and a similar pregnancy rate compared to cycles performed using fresh donor oocytes (Figure 1) [25,26,27,28]. 

**Figure 2:**
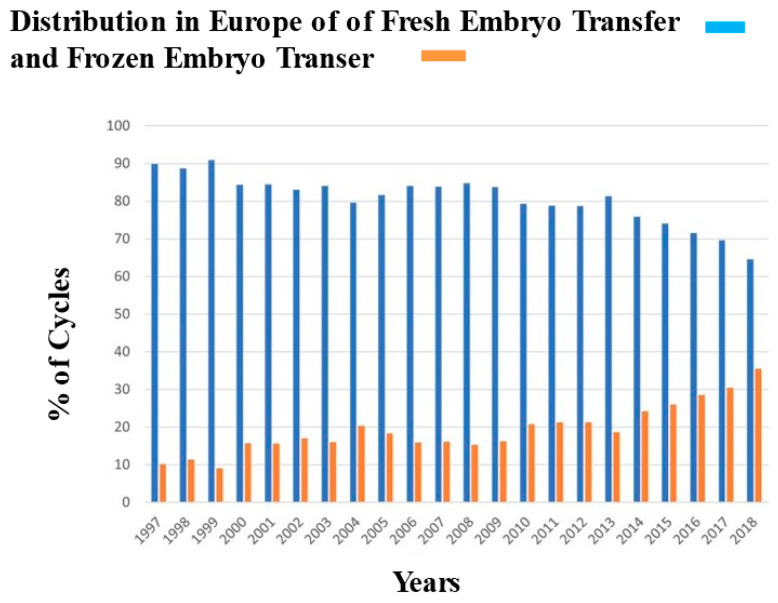
Proportion of fresh and frozen embryo transfers (FETs) performed in Europe (data 1997–2018). Adapted with permission from Wyns and colleagues [2].

In addition to egg freezing cycles, the ART field has assisted in a significant increase in embryo freeze-all cycles. It has been estimated that 600,000 embryos were stored from 2004 to 2013 in the USA alone, and 309,475 FET were completed in 2018 in Europe (Figure 2) [2]. Reasons for embryo cryopreservation are multiple and include storage of surplus embryos following a fresh transfer [29,30], fertility preservation for cancer patients, and pre-implantation genetic testing (Table 1) [31,32]. Additional reasons include abnormalities of the stimulation cycle, including elevated progesterone at the time of trigger (which has been reported to have a negative impact on pregnancy outcomes [33]) or prevention of ovarian hyperstimulation syndrome, a potentially life-threatening complication [34,35,36].

It is important to note that FET is associated with a higher birth weight compared to fresh embryo transfer and no embryo freezing [37,38,39]. A meta-analysis of 26 studies reported that singletons born following freezing and thawing had higher birth weights, were large for gestational age, and the pregnancy had an increased risk of hypertensive disorders [40]. An increased birthweight in ART babies conceived following FET has been reported by several authors [41,42,43,44]. At present, it is unclear whether the vitrification procedure itself, the use of cryoprotective agents (CPAs), the drugs used for endometrial preparation, or parental infertility are responsible for the higher birthweight in offspring. However, since no difference in birth weight has been observed when embryos are transferred in a natural cycle, it is possible that the drugs applied for endometrial preparation might be responsible for that condition [45].

## 3. Cryopreservation and Cryoprotectants

Cryopreservation enables the long-term preservation of tissue or cells at ultra-low temperatures (stored in liquid nitrogen at −196 °C) in a state of suspended animation. This process interrupts all biological activities and maintains cell viability and physiological competency for future use. The first report of a live birth following the transfer of a cryopreserved and thawed embryo was recorded in Australia by Trounson and Mohr in 1983 by the “slow freezing” procedure [46]. Later, in the 1990s, a great advancement in the field was achieved with the introduction of the “vitrification” protocol in Japan and Australia [4,5,47]. Vitrification was rapidly adopted since it achieved better outcomes in terms of gamete and embryo survival and higher pregnancy rates, compared to slow freezing [29,30,48]. Vitrification is performed using a high concentration of CPAs. These agents increase viscosity and inhibit ice crystal formation, inducing the solution to enter a “glassy state” [7]. The success of vitrification is correlated with several factors, such as the temperature in the vitrification and warming steps, which depends on the choice of carrier used (open or closed vitrification) and, most importantly, the concentration and type of CPAs used (Table 2). Regarding the temperature, it has been clearly shown that the warming rate is as important as the cooling rate. Seki and Mazur reported that cryo-damage might also be induced by re-crystallization in the warming step. They examined the relationship between cooling versus warming rates in a mouse model and concluded that a warming rate of at least 3000 °C/min is imperative to obtain an acceptable survival rate above 80% [49]. CPAs play a critical role in the success of cryopreservation and are classified into two categories: Permeating and non-permeating agents. The first group includes small molecular weight compounds (less than 400 Da) that can cross cell membranes and, once inside, protect the cell from cryo-induced damage. Permeating agents include ethylene glycol (EG), dimethyl sulfoxide (DMSO; an amphipathic molecule), propylene glycol or 1,2 propanediol (PG), glycerol (GLY), formamide (FMD), methanol (METH), and butanediol (BD; 2,3-butanediol). DMSO and glycerol are the two most used (Table 3). Non-penetrating CPAs are non-diffusible, normally have a higher molecular weight, and therefore cannot cross the cell membrane. Examples are trehalose, sucrose, glucose, mannitol, galactose, and polyvinylpyrrolidone (PVP). These molecules induce an osmotic gradient that removes water from inside to outside the cell (dehydration), reducing the temperature at which ice starts to form and thus preserving membranes and intracellular structures [50,51].

## 4. Potential Damaging Effects of Cryopreservation

The principal problem that can occur with cryopreservation is the formation of ice crystals. Human embryos and oocytes contain a high content of water, which might be converted into ice, causing irreversible damage and cellular death. This concern was elegantly described by Mazur in 1963 [56]. The sharp reduction of temperature might lead to cold-shock harm and impair the function of several sensible structures located in the oocyte cytoplasm, including membrane permeability, cytoskeleton architecture, and, importantly, the meiotic spindle apparatus [57,58]. The meiotic spindle is a cytoskeletal structure, formed of microtubules and associated proteins [59]. It is considered an indicator of oocyte health; its stability is linked with normal fertilization and is directly responsible for the correct segregation of chromosomes, avoiding errors in chromatin division, accountable for aneuploidies and miscarriage [60]. It is well established that temperature changes can debilitate meiotic spindle stability [61]. At a temperature of 33 °C or lower, the meiotic spindle starts to depolymerize, and only a few minutes of exposure to non-physiologic pH or temperature is sufficient to induce disassembly of the spindle [62]. Several studies on both animals and humans have demonstrated a negative association between temperature, as well as osmolality on normal microtubule disassembly, and spindle alterations [59,60,61,62,63]. Additional impairment following cooling and warming includes premature hardening of the zona pellucida (ZP), which is essential at the time when sperm fertilizes the oocyte. These facts indicate the use of ICSI to fertilize oocytes. However, questions remain concerning the impact of ZP hardening and implantation of the embryo [64]. It is also possible to observe cryo-damage to intracellular organelles, as well as an increased risk of parthenogenetic activation of the oocytes [65]. Oocyte exposure to CPAs might cause ultrastructural modification of the mitochondria and smooth endoplasmic reticulum [66,67]. Animal studies have suggested that oocyte cryopreservation, particularly vitrification, might be associated with increased levels of reactive oxygen species (ROS) and apoptotic events [68,69,70], which might alter the epigenetic mechanisms associated with oocyte competence and future embryo development and viability [70,71]. In particular, DMSO is a known radical scavenger and, as an antioxidant, helps to protect cells from the damage caused by free radicals. However, at normal or decreased levels of ROS, it may restrict cell metabolism by scavenging the electrons needed for ATP production. Therefore, a decrease in DMSO-induced ATP might cause downstream effects that may disrupt cellular function, fetal development, and implantation potential [72,73,74,75]. Finally, over the past few years, several reports have shown the detrimental effects of cryopreservation programs on the epigenetic makeup of the embryo, protein expression, and DNA integrity [76,77,78,79,80], as well as alteration of such genes involved in critical biological processes [79,80,81], inducing an increase in free radical production and apoptosis [81,82,83,84,85]. 

## 5. Epigenetic Changes Occurring during Preimplantation Embryo Development

**Figure 3:**
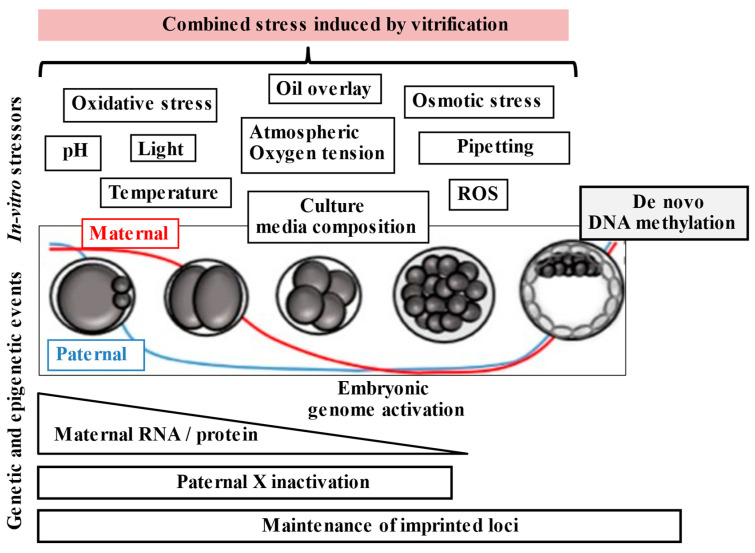
Summary of sensitive genetic and epigenetic events occurring during preimplantation embryo development and when the vitrification procedure is performed. Several stressors exist, and these can act synergistically, causing more negative effects.

In 1942, Conrad Waddington, a biologist at Edinburgh University, was the first to emphasize the importance of environmentally directed changes during the early stages of mammalian embryo development and introduced the term “Epigenetics”. Epigenetics is a gene-regulatory mechanism that leads to heritable changes in gene function that are not associated with changes in DNA sequence [86]. The importance of epigenetics in the ART field is secondary to the fact that epigenetic changes can be caused by different environmental agents and that important epigenetic changes occur during embryo development. There are two epigenetic reprogramming phases. The first resets DNA methylation marks in primordial germ cells (PGCs) when they migrate to the fetal gonadal ridge. The second wave of DNA methylation changes occurs during the early stage of embryo development, following fertilization; the parental genome is actively demethylated, while the maternal genome is passively demethylated with a wave of re-methylation at the blastocyst stage (Figure 3) [87,88,89]. In summary, the epigenome of the preimplantation embryo is highly susceptible to external and internal modifications.

DNA methylation is the most investigated epigenetic process and involves the addition of a methyl group at the 5′ carbon position of the cytosine pyrimidine ring in the context of CG dinucleotide (CpG sites). Those epigenetic modifications are maintained by daughter cells throughout cell divisions by DNA methyltransferases (DNMTs). To date, five different types of DNMTs have been identified: Dnmt1, Dnmt2, Dnmt3a, Dnmt3b, and Dnmt3L [90,91]. DNA methylation is generally correlated with gene silencing, but it is also involved in other regulatory mechanisms such as imprinting or X-chromosome inactivation and silencing of centromeric sequences [90,91,92]. Additional epigenetic regulations comprise post-translational histone modifications, including acetylation, methylation, phosphorylation, and glycosylation ubiquitination [6]. Histone lysine acetylation is particularly important, since it plays a role in cellular differentiation and might be associated with disease processes [93]. This histone modification is regulated by histone acetyltransferases (HATs) and histone deacetylases (HDACs) and is generally associated with transcriptionally active regions of the genome, as it relaxes the chromatin structure, allowing for increased accessibility of the DNA to transcription factors and other regulatory proteins [92,93,94]. Acetylation leads to open chromatin configuration, enhances transcriptional activity, and encourages transcription factor binding to DNA. On the contrary, deacetylation is correlated with transcriptional inactivation and gene silencing [94]. SUMOylation and de-SUMOylation marks indicate the addition and removal of SUMO (small ubiquitin-related modifier) polypeptides on lysine residues [95], which are essential for the occurrence of oocyte maturation, meiotic resumption, and spindle formation [95,96,97]. Finally, another newly identified epigenetic modification is lactylation, affected by cellular lactate levels, which directly stimulates gene transcription [98].

An important subgroup of genes affected by epigenetic regulation are imprinted genes [99,100]. Currently, around 150 genes have been identified in mice, and less than 100 in humans [100]. A list of the current mammalian imprinted genes is available online at [https://www.otago.ac.nz/biochemistry/research/facilities/otago652955.html, accessed on 1 January 2023]. These genes are characterized by a monoallelic expression that is dependent on the parental origin of the allele. The parental imprint is linked to differential epigenetic labeling of parental alleles, and importantly is established during gametogenesis and maintained during the early stage of preimplantation embryo development [101,102,103]. The correct expression of those imprinted genes depicts a critical role in growth and development and are prevalently located in the placenta and brain [104,105,106,107]. Examples include loss of imprinted DNA methylation at the Kvdmr icr, found in ART-conceived children with Beckwith–Wiedemann syndrome (BWS) [108] or gain of methylation because of maternal uniparental disomy on chromosome 7 at the Mest icr in approximately 10% of Silver–Russell Syndrome (SRS) cases, as well as Angelman syndrome (AS) and Prader–Willi syndrome [106,107,108,109,110,111,112,113]. While epigenetic changes can affect the individual, new evidence suggests that there could be a transgenerational transmission of epigenetic information [114]. It is therefore possible that the presence of chemical compounds such as cryoprotective agents could alter the reprogramming machinery and cause long-term risk of disease, as postulated by the Developmental Origin of Health and Disease [115,116,117,118].

## 6. Potential Impact of Vitrification on the Epigenome of Oocytes and Embryos

In the past few years, several research groups have investigated the relationship between vitrification and epigenetic disruption in early embryo development [119]. The most studied molecule and the one most widely used is DMSO. DMSO may impact cellular functions, metabolism, enzyme activities, cell growth, and apoptosis, as well as might induce alterations in microRNAs (miRNA) and epigenetic changes [120,121]. Studies have shown that DMSO has temperature-, time-, and concentration-dependent toxic effects [73,74]. Studies focusing on the effect of DMSO and epigenetic changes have reported that DMSO interferes with the activity of the enzyme DNMT3a, even though the specific mechanism is unknown [119,120,121]. Studies on animal models have shown that following vitrification-warming of mouse oocytes, the expression of the imprinted gene *Kcnq1ot1* decreased significantly [122]. Chen and collaborators reported that following vitrification of mature bovine oocytes, the expression of imprinted genes *Peg10*, *Kcnq1ot1*, and *Xist* in blastocysts obtained by ICSI increased abnormally [123]. The same group in a subsequent publication found that vitrification of mouse MII oocytes affected the expression of the maternally imprinted genes *Peg3*, *Peg10*, and *Igf2r* in oocytes, and maternally imprinted genes *Peg3* and *Peg10* and paternal imprinted gene *Gtl2* in cleavage stage embryos [124]. Another study found that methylation of imprinted genes *H19*, *Peg3*, and *Snrpn* decreased in mouse blastocysts obtained from vitrified mouse oocytes [125]. Comparable results have been reported by other authors, showing a reduction in the overall DNA methylation level in oocytes and early embryos following the vitrification process [126,127]. In summary, animal models suggest that vitrification may affect the normal expression of imprinted genes by changing the DNA methylation level, affecting the regulatory region of those genes (Table 4).

Human studies are limited. A study on the effects of DMSO on the DNA methylation profile in human cardiac microtissues found dysregulation of DNA methylation pathways. Methyltransferase DNMT1, a key factor for the maintenance of DNA methylation, as well as DNMT3A, essential for both de novo and maintenance of DNA methylation, were upregulated, while *TET1*, which plays an important role in active de-methylation, was downregulated [121]. Overall, no or limited changes in DNA methylation and imprinted gene expression were found in human oocytes or embryos following vitrification (Table 4). The imprinted genes *H19* and *Kcnq1ot1* showed no differences in DNA methylation in vitrified oocytes. In this study immature oocytes were donated after egg retrieval, and after vitrification warming were in-vitro matured to MII stage [130]. Liu and colleagues estimated the effects of vitrification on nuclear configuration and global DNA methylation in GV-stage oocytes after vitrification warming and in-vitro maturation to MII stage. They found no significant differences in the distribution of mitochondria and global DNA methylation patterns between the groups. However, the authors reported a significantly higher abnormal configuration of the spindle following vitrification [129]. De Munck reported no significant change in the overall DNA methylation level of in-vitro cultured eight-cell embryos derived from vitrified oocytes [128]. Huo and colleagues, using 16 donated human MII oocytes, observed that a total of 1987 genes were differentially expressed following oocyte vitrification warming compared to fresh mature oocytes and found that about 82% of these genes were downregulated, while 18% were upregulated [136]. Those genes involved in several critical biological processes, such as two meiosis-related genes, *Ncapd2* and *Tubgcp5*, were significantly downregulated following oocyte vitrification. In addition, cryopreservation might induce histone changes in oocytes and preimplantation embryos. Suo and colleagues found that the acetylation status of histone H4 at lysine K12 in mouse oocytes was significantly increased in cryopreserved compared to fresh oocytes [137]. Another study evaluated the consequences of mouse embryo vitrification at two cell stages on specific histone marks (H3K9 methylation and H3K9 acetylation) for the genes *Igf2* and *Oct4.* The authors found no significant difference in the expression level of these genes and their histone marks in vitrified and non-vitrified embryos, while only embryo culture induced changes on these loci [138]. Other pathways that were altered following vitrification included several physiological processes, such as oogenesis, cellular response to heat, microtubule-based processes, methylation, ubiquinone biosynthetic processes, sister chromatid migration, DNA repair, oxidative phosphorylation, and ATP metabolic processes [139,140,141,142]. The authors also investigated the time of storage of vitrified oocytes in nitrogen and found no alteration in gene expression, suggesting that overall, the potential damage resulting from oocyte vitrification might be associated with the cryopreservation process itself rather than the storage [136]. This finding was confirmed by Stigliani and collaborators, who analyzed the gene expression status between surviving warmed oocytes after three and six years of storage in liquid nitrogen and found no differently expressed genes [143]. The effects of the length of freezing embryos in liquid nitrogen on thawing survival, blastocyst viability, and implantation were recently investigated by Yan and colleagues, who evaluated pregnancy outcomes following different lengths of storage (from less than three years up to 10 years). The authors found a reduced survival rate for blastocysts that were stored for longer than six years. Similarly, clinical pregnancy and live birth rates were significantly decreased in blastocysts stored for more than six years compared with the group frozen for less than three years. No difference was reported in the rates of miscarriage and ectopic pregnancy [144]. In summary, while epigenetic changes in oocytes and embryos following cryopreservation exist, their significance and clinical consequences remain to be fully elucidated [145,146,147,148]. Future studies are needed to clarify this important issue [148].

## 7. Potential Impact of Vitrification on the Epigenome Spermatozoa

Sperm cryopreservation is an essential component of ART that has wide clinical applications while being critical for cancer patients to protect their fertility before receiving chemotherapy or radiotherapy [149,150,151]. Cryopreservation of human sperm has been practiced for more than 50 years [151]. In the past decade, sperm vitrification has been shown to achieve a higher survival rate and reduced sperm DNA damage compared to slow-freezing protocols [151,152,153,154]. Several studies have investigated the impact of sperm cryopreservation on epigenetic markers, including DNA methylation, histone modification, and non-coding RNA molecules [155,156,157,158,159,160,161,162]. De Mello and co-authors investigated the effect of CPA, methanol, ethyl glycol, and glycerol dimethylsulfoxide on DNA methylation of *Colossoma macropomum* sperm and embryo evolution and found that the cryoprotectants investigated induced an overall reduction in DNA methylation levels in spermatozoa, and also caused a significant delay in embryonic development [163]. In contrast, a study by Depince and collaborators reported that DNA methylation of zebrafish spermatozoa significantly increased after cryopreservation with methanol [164]. Salehi and colleagues studied DNA methylation and histone modification, as well as cellular features, including membrane integrity, mitochondria activity and apoptosis, and fertility potential, of rooster semen before and after cryopreservation. The results showed that cryopreservation leads to significantly reduced values of the parameters examined when correlated with fresh samples. Furthermore, there was a significant reduction in H3K9 acetylation and H3K4 methylation compared to the fresh samples [165]. Another study showed that cryoprotectant and freezing–thawing protocols significantly increased global DNA methylation levels in ram spermatozoa [166]. Additionally, a study on humans by Khosravizadeh and co-authors investigated the effects of cryopreservation on DNA methylation in promoter regions of the SNURF–SNRPN and UBE3A imprinted genes, PWS-ICR, and AS-ICR in the chromosome 15q11–q13 region [167]. The authors reported the cryopreservation method to be safe concerning DNA methylation in the chromosome 15q11–q13 region. They found that exposure to cryoprotectants had no significant effect on ROS levels and DNA fragmentation. Neither cryopreservation nor exposure to cryoprotectant significantly affected DNA methylation of the selected gene regions. However, DNA fragmentation had a positive correlation with DNA methylation of AS-ICR [167]. Different mechanisms could lead to epigenetic changes following cryopreservation. First, cryoprotectant agents could be responsible. For example, CPA, a widely used agent for sperm cryopreservation, is cytotoxic and can harm sperm cells, causing osmotic injury and physiological alterations and potentially influencing the epigenetic state of sperm cells indirectly [149,154,155,156,157]. Second, raising the level of ROS during the freezing–thawing process [156,159,160,161] might induce site-specific hypermethylation through either the upregulation of DNA methyltransferases (DNMTs) or the formation of new DNMT-including complexes [158,159]. It is important to emphasize that sperm epigenetic changes could be secondary to additional factors, including sperm manipulation alone or patient characteristics [166,167,168,169,170]. For example, it is well known that oligospermic men have more epigenetic changes than normospermic men [160,161,162]. However, the number of studies currently available on the topic is still limited. Given the relatively low number of studies conducted using human spermatozoa, additional multicenter studies utilizing the same cryopreservation protocols and DNA methylation analysis are needed to clarify the issue.

## 8. Conclusions 

In the last decade, advancements made in the field of cryobiology have contributed to the increased success of ART. However, concerns about the association between cryopreservation and alteration in epigenetic reprogramming exist. This is relevant, given the association between epigenetic changes and future offspring health. Unfortunately, evidence is lacking, and the number of published reports is limited. Future studies and utilization of novel technologies (such as single-cell sequencing and epigenomics) are needed to fully assess the potential epigenetic aberration that occurs at the time of oocytes or embryo cryopreservation, in order to improve its safety and efficacy in ART.

## Figures and Tables

**Table 1:** Main indications for the application of human embryo cryopreservation.

	Embryo Cryopreservation in ART Practice
Preimplantation genetic testing	Genetic assessment is facilitated by the opportunity to utilize the cryopreservation method to store embryos to be transferred in a future cycle, and to overcome the time interval between the blastocyst biopsy and genetic result
Avoiding ovarian hyperstimulation syndrome (OHSS)	When a fresh embryo transfer cannot be performed due to the risk of OHSS, embryos might be cryopreserved and used in a future cycle
Increasing the policy of elective single embryo transfer (eSET)	The cryopreservation of surplus embryos is considered a valid method to reduce the number of embryos transferred during a fresh cycle and to thus minimize the risk of multiple pregnancies and to increase the policy of eSET—as well as to reduce the need for repeated stimulation cycles
Embryo freezing for cancer patients	In women with a stable partner about to go through gonadotoxic/chemotherapy treatment for cancer
Elevated progesterone or other conditions, such as endometriosis	Elevated progesterone in the late follicular phase has a negative impact on pregnancy outcomes; or other conditions and medical pathology that might affect fertility

**Table 2:** Membrane permeability coefficient of some cryoprotectants (Times 10^−5^ cm/s).

Cryoprotectant	Red Blood Cells at 4 °C Study Reference [52]	Sperm Cells at 22 °C Study Reference [53]	Oocytes at 22 °C Study Reference [54]
Methanol	11.35	N/A	N/A
Formamide	8.05	N/A	N/A
Ethylene glycol	3.38	13.2	1.95
Dimethyl sulfoxide	1.30	1.33	2.60
Propylene glycol	1.79	3.83	3.83
Glycerol	0.58	3.50	Low

**Table 3:** Minimal concentration required to vitrify (C-Vit) for some permeating cryoprotectants at a pressure of 1 atmosphere according to Fahy and colleagues 1984 [55].PG, propylene glycol; DMSO, dimethyl sulfoxide; EG, ethylene glycol; GLY, glycerol.

Cryoprotectants	Concentration Required to Vitrify (C-Vit) %/Volume
DMSO	49–50
PG	43.5
EG	55
GLY	65

**Table 4:** Summary of both human and animal studies showing the effects of vitrification on DNA methylation and histone modifications. GV, oocyte at germinal vesicle stage; MII, oocyte at metaphase II stage; IVM, in vitro maturation; 5hmC, 5-hydroxymethylCytosine; 5mC, 5-methylCytosine; DMR, differentially methylated regions.

Study [Ref.]	Materials: Human or Animal	Oocytes or Embryo Analyzed (*n*)	Technology of Assessment	Studied Sequences or Genes	Main Findings
De Munck et al. [128]	(Human) Mature (MII) donated oocytes	31 embryos (Day 3) from 17 fresh oocytes and 14 after vitrification	Immunofluorescence (5mC, 5hmC)	Global Analysis	No differences in fluorescence intensities between embryos from fresh and vitrified oocytes
Liu et al. [129]	(Human) Vitrified mature oocytes (MII), and MII from GV matured in-vitro	56 in vivo MII, 106 MII from GV matured in-vitro, 122 MII from vitrified GV	Immunofluorescence (5mC)	Global analysis	No significant differences in fluorescence intensities between groups
Al-Khtib et al. [130]	(Human) GV oocytes donated for research and IVM to MII	77 MII after IVM from 184 vitrified GV stage, and 85 MII from 120 fresh GV	Pyrosequencing	Methylation profile of *H19* and *KCNQ1OT1*, *H19DMR* and *KvDMR1*	Oocyte vitrification at the GV stage does not affect the methylation profiles of *H19-DMR* and *KvDMR1*
Cantatore et al. [131]	(Mouse) Cleavage stage embryos and blastocysts from vitrified MII oocytes	Two-cell embryos and blastocysts from vitrified oocytes	q-PCR	*Igf2r* and *Gtl2*	No significant differences observed
Zhao et al. [126]	(Bovine) Oocytes	Vitrified MII oocytes matured in-vitro	Single-cell whole-genome methylation sequencing	Global analysis	*Peg3* methylation level significantly decreased in the derived blastocysts
Chen et al. [124]	(Mouse) Oocytes	MII oocytes and two-cell embryos	q-PCR and bisulfite sequencing	*Gtl2*, *H19*, *Igf2*, *Peg3*, *Peg10*, *Igf2r*	*Peg3*, *Peg10*, and *Igf2r* were significantly different in MII oocytes and two-cell embryos after vitrification
Chen et al. [123]	(Bovine) Oocytes	Vitrified MII oocytes matured in vitro	q-PCR	*Peg3*, *Peg10*, *Kcnq1ot1*, *Xist*, *Igf2r*	*Peg10*, *Kcnq1ot1*, and *Xist* significantly increased after vitrification
Cheng et al. [76]	(Mouse) Blastocysts	Blastocysts from vitrified MII oocytes	Bisulfite sequencing	*H19*, *Peg3*, *Snrpn*	No significant differences in oocytes; decrease in blastocysts after oocyte vitrification
Ma et al. [122]	(Mouse) Oocytes	Mature metaphase II oocytes	WGBS combined with RNA-seq	Global analysis	*Kcnq1ot1* was significantly downregulated in the vitrified oocytes
Jahangiri et al. [132]	(Mouse) Embryos	Mouse blastocysts from vitrified two-cell embryos	q-PCR	*H3*, *H19* and *Mest*	The expression level of the chosen imprinted genes increased significantly in experimental groups compared to in vivo blastocysts
Movahed et al. [133]	(Mouse) Embryos	Mouse blastocysts from vitrified two-cell embryos	q-PCR	*Gtl2* and *Dlk1*	*Gtl2* was downregulated and *Dlk1* was upregulated after vitrification
Barberet et al. [134]	(Human) Placenta	Human placenta	Pyrosequencing and q-PCR	*H19*, *IGF2*, *KCNQ1OT1* *SNURF*	The placental DNA methylation levels of *H19/IGF2* were lower in the fresh embryo transfer group than in the control (*H19/IGF2*-seq1) and frozen embryo transfer (*H19/IGF2*-seq2) groups
Yao et al. [135]	(Human) Placenta	Human placenta obtained from vitrified embryos	q-PCR, Western blotting, and pyrosequencing	*SNRPN*	The expression level of SNRPN increased after vitrification

## Data Availability

No data is available.

## References

[1] De Geyter C., Calhaz-Jorge C., Kupka M.S., Wyns C., Mocanu E., Motrenko T., Scaravelli G., Smeenk J., Vidakovic S., Goossens V. (2020). ART in Europe, 2015: Results generated from European registries by ESHRE. Hum. Reprod. Open.

[2] Wyns C., De Geyter C., Calhaz-Jorge C., Kupka M.S., Motrenko T., Smeenk J., Bergh C., Tandler-Schneider A., Rugescu I.A., Goossens V. (2022). ART in Europe, 2018: Results generated from European registries by ESHRE. Hum. Reprod. Open.

[3] Chen C. (1986). Pregnancy after human oocyte cryopreservation. Lancet.

[4] Kuwayama M., Vajta G., Kato O., Leibo S.P. (2005). Highly efficient vitrification method for cryopreservation of human oocytes. Reprod. Biomed. Online.

[5] Rienzi L., Gracia C., Maggiulli R., LaBarbera A.R., Kaser D.J., Ubaldi F.M., Vanderpoel S., Racowsky C. (2017). Oocyte, embryo and blastocyst cryopreservation in art: Systematic review and meta-analysis comparing slow-freezing versus vitrification to produce evidence for the development of global guidance. Hum. Reprod. Update.

[6] Potdar N., Gelbaya T.A., Nardo L.G. (2014). Oocyte vitrification in the 21st century and post-warming fertility outcomes: A systematic review and meta-analysis. Reprod. Biomed. Online.

[7] Hubel A., Spindler R., Skubitz A. (2014). Storage of Human Biospecimens: Selection of the Optimal Storage Temperature. Biopreserv. Biobank..

[8] Hart R., Norman R.J. (2013). The longer-term health outcomes for children born as a result of IVF treatment: Part I–General health outcomes. Hum. Reprod. Update.

[9] Ventura-Juncá P., Irarrázaval I., Rolle A.J., Gutiérrez J.I., Moreno R.D., Santos M.J. (2015). In vitro fertilization (IVF) in mammals: Epigenetic and developmental alterations. Scientific and bioethical implications for IVF in humans. Biol. Res..

[10] Vrooman L.A., Bartolomei M.S. (2017). Can assisted reproductive technologies cause adult-onset disease? Evidence from human and mouse. Reprod. Toxicol..

[11] Hirasawa R., Feil R. (2010). Genomic imprinting and human disease. Essays Biochem..

[12] Smith Z.D., Chan M.M., Humm K.C., Karnik R., Mekhoubad S., Regev A., Eggan K., Meissner A. (2014). DNA methylation dynamics of the human preimplantation embryo. Nature.

[13] Chronopoulou E., Raperport C., Sfakianakis A., Srivastava G., Homburg R. (2021). Elective oocyte cryopreservation for age-related fertility decline. J. Assist. Reprod. Genet..

[14] Cobo A., Garcia-Velasco J., Domingo J., Pellicer A., Remohí J. (2018). Elective and Onco-fertility preservation: Factors related to IVF outcomes. Hum. Reprod..

[15] Nasab S., Ulin L., Nkele C., Shah J., Abdallah M.E., Sibai B.M. (2020). Elective egg freezing: What is the vision of women around the globe?. Future Sci. OA.

[16] Seyhan A., Akin O.D., Ertaş S., Ata B., Yakin K., Urman B. (2021). A Survey of Women Who Cryopreserved Oocytes for Non-medical Indications (Social Fertility Preservation). Reprod. Sci..

[17] Sung H., Ferlay J., Siegel R.L., Laversanne M., Soerjomataram I., Jemal A., Bray F. (2021). Global Cancer Statistics 2020: GLOBOCAN Estimates of Incidence and Mortality Worldwide for 36 Cancers in 185 Countries. CA Cancer J. Clin..

[18] Stearns V., Schneider B., Henry N.L., Hayes D.F., Flockhart D.A. (2006). Breast cancer treatment and ovarian failure: Risk factors and emerging genetic determinants. Nat. Rev. Cancer.

[19] Adams D., A Clark R., Davies M., de Lacey S. (2015). A meta-analysis of neonatal health outcomes from oocyte donation. J. Dev. Orig. Health Dis..

[20] Trounson A., Leeton J., Besanko M., Wood C., Conti A. (1983). Pregnancy established in an infertile patient after transfer of a donated embryo fertilised in vitro. BMJ.

[21] Sauer M.V., Kavic S.M. (2006). Oocyte and embryo donation 2006: Reviewing two decades of innovation and controversy. Reprod. Biomed. Online.

[22] Kawwass J.F., Eyck P.T., Sieber P., Hipp H.S., Van Voorhis B. (2021). More than the oocyte source, egg donors as patients: A national picture of United States egg donors. J. Assist. Reprod. Genet..

[23] Cobo A., Garrido N., Pellicer A., Remohí. J. (2015). Six years’ experience in ovum donation using vitrified oocytes: Report of cumulative outcomes, impact of storage time, and development of a predictive model for oocyte survival rate. Fertil. Steril..

[24] Cobo A., Meseguer M., Remoh J., Pellicer A. (2010). Use of cryo-banked oocytes in an ovum donation programme: A prospective, randomized, controlled, clinical trial. Hum. Reprod..

[25] Debrock S., Peeraer K., Gallardo E.F., De Neubourg D., Spiessens C., D’Hooghe T. (2015). Vitrification of cleavage stage day 3 embryos results in higher live birth rates than conventional slow freezing: A RCT. Hum. Reprod..

[26] Rienzi L., Cimadomo D., Maggiulli R., Vaiarelli A., Dusi L., Buffo L., Amendola M.G., Colamaria S., Giuliani M., Bruno G. (2020). Definition of a clinical strategy to enhance the efficacy, efficiency and safety of egg donation cycles with imported vitrified oocytes. Hum. Reprod..

[27] Sciorio R., Antonini E., Engl B. (2021). Live birth and clinical outcome of vitrification-warming donor oocyte programme: An experience of a single IVF unit. Zygote.

[28] Rienzi L., Romano S., Albricci L., Maggiulli R., Capalbo A., Baroni E., Colamaria S., Sapienza F., Ubaldi F. (2009). Embryo development of fresh ‘versus’ vitrified metaphase II oocytes after ICSI: A prospective randomized sibling-oocyte study. Hum. Reprod..

[29] Sciorio R., Thong K., Pickering S.J. (2018). Single blastocyst transfer (SET) and pregnancy outcome of day 5 and day 6 human blastocysts vitrified using a closed device. Cryobiology.

[30] Sciorio R., Thong K.J., Pickering S.J. (2019). Increased pregnancy outcome after day 5 versus day 6 transfers of human vitrified-warmed blastocysts. Zygote.

[31] Sciorio R., Anderson R.A. (2019). Fertility preservation and preimplantation genetic assessment for women with breast cancer. Cryobiology.

[32] Somigliana E., Viganò P., Filippi F., Papaleo E., Benaglia L., Candiani M., Vercellini P. (2015). Fertility preservation in women with endometriosis: For all, for some, for none?. Hum. Reprod..

[33] Santos-Ribeiro S., Polyzos N., Haentjens P., Smitz J., Camus M., Tournaye H., Blockeel C. (2014). Live birth rates after IVF are reduced by both low and high progesterone levels on the day of human chorionic gonadotrophin administration. Hum. Reprod..

[34] Groenewoud E.R., Cohlen B.J., Macklon N.S. (2018). Programming the endometrium for deferred transfer of cryopreserved embryos: Hormone replacement versus modified natural cycles. Fertil. Steril..

[35] Sullivan E.A., Wang Y.A., Hayward I., Chambers G.M., Illingworth P., McBain J., Norman R.J. (2012). Single embryo transfer reduces the risk of perinatal mortality, a population study. Hum. Reprod..

[36] Sciorio R., Esteves S.C. (2019). Clinical utility of freeze-all approach in ART treatment: A mini-review. Cryobiology.

[37] Belva F., Bonduelle M., Roelants M., Verheyen G., Van Landuyt L. (2016). Neonatal health including congenital malformation risk of 1072 children born after vitrified embryo transfer. Hum. Reprod..

[38] Hwang S.S., Dukhovny D., Gopal D., Cabral H., Diop H., Coddington C.C., Stern J.E. (2019). Health outcomes for Massachusetts infants after fresh versus frozen embryo transferr. Fertil. Steril..

[39] Ainsworth A.J., Wyatt M.A., Shenoy C.C., Hathcock M., Coddington C.C. (2019). Fresh versus frozen embryo transfer has no effect on childhood weight. Fertil. Steril..

[40] Maheshwari A., Pandey S., Raja E.A., Shetty A., Hamilton M., Bhattacharya S. (2017). Is frozen embryo transfer better for mothers and babies? Can cumulative meta-analysis provide a definitive answer?. Hum. Reprod. Update.

[41] Maheshwari A., Raja E.A., Bhattacharya S. (2016). Obstetric and perinatal outcomes after either fresh or thawed frozen embryo transfer: An analysis of 112,432 singleton pregnancies recorded in the Human Fertilisation and Embryology Authority anonymized dataset. Fertil. Steril..

[42] Sazonova A., Källen K., Thurin-Kjellberg A., Wennerholm U.-B., Bergh C. (2012). Obstetric outcome in singletons after in vitro fertilization with cryopreserved/thawed embryos. Hum. Reprod..

[43] Pelkonen S., Koivunen R., Gissler M., Nuojua-Huttunen S., Suikkari A.-M., Hydén-Granskog C., Martikainen H., Tiitinen A., Hartikainen A.-L. (2010). Perinatal outcome of children born after frozen and fresh embryo transfer: The Finnish cohort study 1995–2006. Hum. Reprod..

[44] Pinborg A., Henningsen A.A., Loft A., Malchau S.S., Forman J., Andersen A.N. (2014). Large baby syndrome in singletons born after frozen embryo transfer (FET): Is it due to maternal factors or the cryotechnique?. Hum. Reprod..

[45] von Versen-Höynck F., Narasimhan P., Tierney E.S.S., Martinez N., Conrad K.P., Baker V.L., Winn V.D. (2019). Absent or Excessive Corpus Luteum Number Is Associated with Altered Maternal Vascular Health in Early Pregnancy. Hypertension.

[46] Trounson A., Mohr L. (1983). Human pregnancy following cryopreservation, thawing and transfer of an eight-cell embryo. Nature.

[47] Mukaida T., Wada S., Takahashi K., Pedro P., An T., Kasai M. (1998). Vitrification of human embryos based on the assessment of suitable conditions for 8-cell mouse embryos. Hum. Reprod..

[48] Li Z., Wang A.Y., Ledger W., Edgar D.H., Sullivan E.A. (2014). Clinical outcomes following cryopreservation of blastocysts by vitrification or slow freezing: A population-based cohort study. Hum. Reprod..

[49] Seki S., Mazur P. (2009). The dominance of warming rate over cooling rate in the survival of mouse oocytes subjected to a vitrification procedure. Cryobiology.

[50] Karlsson J.O., Toner M. (1996). Long-term storage of tissues by cryopreservation: Critical issues. Biomaterials.

[51] Fuller B.J. (2004). Cryoprotectants: The essential antifreezes to protect life in the frozen state. CryoLetters.

[52] Naccache P., Sha’Afi R.I. (1973). Patterns of Nonelectrolyte Permeability in Human Red Blood Cell Membrane. J. Gen. Physiol..

[53] Gilmore J.A., Liu J., Gao D.Y., Critser J.K. (1997). Determination of optimal cryoprotectants and procedures for their addition and removal from human spermatozoa. Hum. Reprod..

[54] Van den Abbeel E., Schneider U., Liu J., Agca Y., Critser J.K., Van Steirteghem A. (2007). Osmotic responses and tolerance limits to changes in external osmolalities, and oolemma permeability characteristics, of human in vitro matured MII oocytes. Hum. Reprod..

[55] Fahy G.M., Macfarlane D.R., Angell C.A., Meryman H.T. (1984). Vitrification as an approach to cryopreservation. Cryobiology.

[56] Mazur P. (1963). Kinetics of Water Loss from Cells at Subzero Temperatures and the Likelihood of Intracellular Freezing. J. Gen. Physiol..

[57] Smith G.D., Silva E.S.C.A. (2004). Developmental consequences of cryopreservation of mammalian oocytes and embryos. Reprod. Biomed. Online.

[58] Best B.P. (2015). Cryoprotectant toxicity: Facts, issues, and questions. Rejuvenation Res..

[59] Dal Canto M., Guglielmo M.C., Mignini Renzini M., Fadini R., Moutier C., Merola M., De Ponti E., Coticchio G. (2017). Dysmorphic patterns are associated with cytoskeletal alterations in human oocytes. Hum. Reprod..

[60] Feuer S., Rinaudo P. (2012). Preimplantation stress and development. Birth Defects Res. C Embryo Today.

[61] Wang W.-H., Meng L., Hackett R.J., Oldenbourg R., Keefe D.L. (2002). Rigorous thermal control during intracytoplasmic sperm injection stabilizes the meiotic spindle and improves fertilization and pregnancy rates. Fertil. Steril..

[62] Montag M., van der Ven H. (2008). Symposium: Innovative techniques in human embryo viability assessment. Oocyte assessment and embryo viability prediction: Birefringence imaging. Reprod. Biomed. Online.

[63] Pickering S.J., Braude P.R., Johnson M.H., Cant A., Currie J. (1990). Transient cooling to room temperature can cause irreversible disruption of the meiotic spindle in the human oocyte. Fertil. Steril..

[64] Larman M.G., Sheehan C.B., Gardner D.K. (2006). Calcium-free vitrification reduces cryoprotectant-induced zona pellucida hardening and increases fertilization rates in mouse oocytes. Reproduction.

[65] Gook D.A., Osborn S.M., Johnston W.I. (1995). Parthenogenetic activation of human oocytes following cryopreservation using 1,2-propanediol. Hum. Reprod..

[66] Gualtieri R., Iaccarino M., Mollo V., Prisco M., Iaccarino S., Talevi R. (2009). Slow cooling of human oocytes: Ultrastructural injuries and apoptotic status. Fertil. Steril..

[67] Jones A., VAN Blerkom J., Davis P., Toledo A.A. (2004). Cryopreservation of metaphase II human oocytes effects mitochondrial membrane potential: Implications for developmental competence. Hum. Reprod..

[68] Kohaya N., Fujiwara K., Ito J., Kashiwazaki N. (2013). Generation of Live Offspring from Vitrified Mouse Oocytes of C57BL/6J Strain. PLoS ONE.

[69] Zhao X.-M., Hao H.-S., Du W.-H., Zhao S.-J., Wang H.-Y., Wang N., Wang D., Liu Y., Qin T., Zhu H.-B. (2015). Melatonin inhibits apoptosis and improves the developmental potential of vitrified bovine oocytes. J. Pineal Res..

[70] Christou-Kent M., Dhellemmes M., Lambert E., Ray P.F., Arnoult C. (2020). Diversity of RNA-Binding Proteins Modulating Post-Transcriptional Regulation of Protein Expression in the Maturing Mammalian Oocyte. Cells.

[71] Sendzikaite G., Kelsey G. (2019). The role and mechanisms of DNA methylation in the oocyte. Essays Biochem..

[72] Yu Z.W., Quinn P.J. (1994). Dimethyl sulphoxide: A review of its applications in cell biology. Biosci. Rep..

[73] Hunt C.J. (2011). Cryopreservation of Human Stem Cells for Clinical Application: A Review. Transfus. Med. Hemother..

[74] Marks P.A., Breslow R. (2007). Dimethyl sulfoxide to vorinostat: Development of this histone deacetylase inhibitor as an anticancer drug. Nat. Biotechnol..

[75] Zorov D.B., Juhaszova M., Sollott S.J. (2014). Mitochondrial Reactive Oxygen Species (ROS) and ROS-Induced ROS Release. Physiol. Rev..

[76] Cheng K.R., Fu X.W., Zhang R.N., Jia G.X., Hou Y.P., Zhu S.E. (2014). Effect of oocyte vitrification on deoxyribonucleic acid methylation of H19, Peg3, and Snrpn differentially methylated regions in mouse blastocysts. Fertil. Steril..

[77] Kader A., Agarwal A., Abdelrazik H., Sharma R.K., Ahmady A., Falcone T. (2009). Evaluation of post-thaw DNA integrity of mouse blastocysts after ultrarapid and slow freezing. Fertil. Steril..

[78] Kopeika J., Thornhill A., Khalaf Y. (2015). The effect of cryopreservation on the genome of gametes and embryos: Principles of cryobiology and critical appraisal of the evidence. Hum. Reprod. Update.

[79] Diaferia G.R., Dessì S.S., DeBlasio P., Biunno I. (2008). Is stem cell chromosomes stability affected by cryopreservation conditions?. Cytotechnology.

[80] Katkov I.I., Kim M.S., Bajpai R., Altman Y.S., Mercola M., Loring J.F., Terskikh A.V., Snyder E.Y., Levine F. (2006). Cryopreservation by slow cooling with DMSO diminished production of Oct-4 pluripotency marker in human embryonic stem cells. Cryobiology.

[81] Wagh V., Meganathan K., Jagtap S., Gaspar J.A., Winkler J., Spitkovsky D., Hescheler J., Sachinidis A. (2011). Effects of cryopreservation on the transcriptome of human embryonic stem cells after thawing and culturing. Stem Cell Rev. Rep..

[82] Xu X., Cowley S., Flaim C.J., James W., Seymour L., Cui Z. (2010). The roles of apoptotic pathways in the low recovery rate after cryopreservation of dissociated human embryonic stem cells. Biotechnol. Prog..

[83] Li M., Feng C., Gu X., He Q., Wei F. (2017). Effect of cryopreservation on proliferation and differentiation of periodontal ligament stem cell sheets. Stem Cell Res. Ther..

[84] Estill M.S., Bolnick J.M., Waterland R.A., Bolnick A.D., Diamond M.P., Krawetz S.A. (2016). Assisted reproductive technology alters deoxyribonucleic acid methylation profiles in bloodspots of newborn infants. Fertil. Steril..

[85] Cui M., Dong X., Lyu S., Zheng Y., Ai J. (2021). The Impact of Embryo Storage Time on Pregnancy and Perinatal Outcomes and the Time Limit of Vitrification: A Retrospective Cohort Study. Front. Endocrinol..

[86] Waddington C.H. (2012). The epigenotype. Int J. Epidemiol..

[87] Mak W., Weaver J.R., Bartolomei M.S. (2010). Is ART changing the epigenetic landscape of imprinting?. Anim. Reprod..

[88] Marcho C., Cui W., Mager J. (2015). Epigenetic dynamics during preimplantation development. Reproduction.

[89] Skinner M.K. (2011). Environmental epigenomics and disease susceptibility. EMBO Rep..

[90] Rivera C.M., Ren B. (2013). Mapping Human Epigenomes. Cell.

[91] Gujar H., Weisenberger D.J., Liang G. (2019). The Roles of Human DNA Methyltransferases and Their Isoforms in Shaping the Epigenome. Genes.

[92] Goldberg A.D., Allis C.D., Bernstein E. (2007). Epigenetics: A Landscape Takes Shape. Cell.

[93] Gallinari P., Di Marco S., Jones P., Pallaoro M., Steinkühler C. (2007). HDACs, histone deacetylation and gene transcription: From molecular biology to cancer therapeutics. Cell Res..

[94] Liu Y., Lu C., Yang Y., Fan Y., Yang R., Liu C.-F., Korolev N., Nordenskiöld L. (2011). Influence of Histone Tails and H4 Tail Acetylations on Nucleosome–Nucleosome Interactions. J. Mol. Biol..

[95] Schatten H., Sun Q.Y., Sutovsky P. (2014). Posttranslationally Modified Tubulins and other Cytoskeletal Proteins: Their Role in Gametogenesis, Oocyte Maturation, Fertilization and Pre-Implantation Embryo Development. Posttranslational Protein Modifications in the Reproductive System.

[96] Feitosa W.B., Hwang K., Morris P.L. (2017). Temporal and SUMO-specific SUMOylation contribute to the dynamics of Polo-like kinase 1 (PLK1) and spindle integrity during mouse oocyte meiosis. Dev. Biol..

[97] Rodriguez A., Briley S.M., Patton B.K., Tripurani S.K., Rajapakshe K., Coarfa C., Rajkovic A., Andrieux A., Dejean A., Pangas S.A. (2019). Loss of the E2 SUMO-conjugating enzyme Ube2i in oocytes during ovarian folliculogenesis causes infertility in mice. Development.

[98] Zhang D., Tang Z., Huang H., Zhou G., Cui C., Weng Y., Liu W., Kim S., Lee S., Perez-Neut M. (2019). Metabolic regulation of gene expression by histone lactylation. Nature.

[99] Skaar D.A., Li Y., Bernal A.J., Hoyo C., Murphy S.K., Jirtle R.L. (2012). The human imprintome: Regulatory mechanisms, methods of ascertainment, and roles in disease susceptibility. ILAR J..

[100] Glaser R.L. (2006). The imprinted gene and parent-of-origin effect database now includes parental origin of de novo mutations. Nucleic Acids Res..

[101] Li X. (2013). Genomic imprinting is a parental effect established in mammalian germ cells. Curr. Top Dev. Biol..

[102] Das R., Lee Y.K., Strogantsev R., Jin S., Lim Y.C., Ng P.Y., Lin X.M., Chng K., Yeo G.S., Ferguson-Smith A.C. (2013). DNMT1 and AIM1 Imprinting in human placenta revealed through a genome-wide screen for allele-specific DNA methylation. BMC Genom..

[103] Thamban T., Agarwaal V., Khosla S. (2020). Role of genomic imprinting in mammalian development. J. Biosci..

[104] Kalish J.M., Jiang C., Bartolomei M.S. (2014). Epigenetics and imprinting in human disease. Int. J. Dev. Biol..

[105] Eggermann T., de Nanclares G.P., Maher E.R., Temple I.K., Tümer Z., Monk D., Mackay D.J.G., Grønskov K., Riccio A., Linglart A. (2015). Imprinting disorders: A group of congenital disorders with overlapping patterns of molecular changes affecting imprinted loci. Clin. Epigenet..

[106] Hiura H., Okae H., Chiba H., Miyauchi N., Sato F., Sato A., Arima T. (2014). Imprinting methylation errors in ART. Reprod. Med. Biol..

[107] Lazaraviciute G., Kauser M., Bhattacharya S., Haggarty P., Bhattacharya S. (2014). A systematic review and meta-analysis of DNA methylation levels and imprinting disorders in children conceived by IVF/ICSI compared with children conceived spontaneously. Hum. Reprod. Update.

[108] Azzi S., Habib A.W., Netchine I. (2014). Beckwith-Wiedemann and Russell-Silver Syndromes: From New Molecular Insights to the Comprehension of Imprinting Regulation. Curr. Opin. Endocrinol. Diabetes Obes..

[109] Mabb A.M., Judson M.C., Zylka M.J., Philpot B.D. (2011). Angelman Syndrome: Insights into Genomic Imprinting and Neurodevelopmental Phenotypes. Trends Neurosci..

[110] Cassidy S.B., Schwartz S., Miller J.L., Driscoll D.J. (2012). Prader-Willi Syndrome. Genet. Med..

[111] DeBaun M.R., Niemitz E.L., Feinberg A.P. (2003). Association of In Vitro Fertilization with Beckwith-Wiedemann Syndrome and Epigenetic Alterations of LIT1 and H19. Am. J. Hum. Genet..

[112] Gicquel C., Gaston V., Mandelbaum J., Siffroi J.-P., Flahault A., Le Bouc Y. (2003). In Vitro Fertilization May Increase the Risk of Beckwith-Wiedemann Syndrome Related to the Abnormal Imprinting of the KCNQ1OT Gene. Am. J. Hum. Genet..

[113] Halliday J., Oke K., Breheny S., Algar E., Amor D.J. (2004). Beckwith-Wiedemann Syndrome and IVF: A Case-Control Study. Am. J. Hum. Genet..

[114] Russo V.E.A., Martienssen R.A., Riggs A.D. (1996). Epigenetic Mechanisms of Gene Regulation.

[115] Klose R.J., Bird A.P. (2006). Genomic DNA methylation: The mark and its mediators. Trends Biochem. Sci..

[116] Bannister A.J., Kouzarides T. (2011). Regulation of chromatin by histone modifications. Cell Res..

[117] Faulk C., Dolinoy D.C. (2011). Timing is everything: The when and how of environmentally induced changes in the epigenome of animals. Epigenetics.

[118] Weaver J.R., Susiarjo M., Bartolomei M.S. (2009). Imprinting and epigenetic changes in the early embryo. Mamm. Genome.

[119] Iwatani M., Ikegami K., Kremenska Y., Hattori N., Tanaka S., Yagi S., Shiota K. (2006). Dimethyl Sulfoxide Has an Impact on Epigenetic Profile in Mouse Embryoid Body. Stem Cells.

[120] Santos N.C., Figueira-Coelho J., Martins-Silva J., Saldanha C. (2003). Multidisciplinary utilization of dimethyl sulfoxide: Pharmacological, cellular, and molecular aspects. Biochem. Pharmacol..

[121] Verheijen M., Lienhard M., Schrooders Y., Clayton O., Nudischer R., Boerno S., Timmermann B., Selevsek N., Schlapbach R., Gmuender H. (2019). DMSO induces drastic changes in human cellular processes and epigenetic landscape in vitro. Sci. Rep..

[122] Ma Y., Long C., Liu G., Bai H., Ma L., Bai T., Zuo Y., Li S. (2021). WGBS combined with RNA-seq analysis revealed that Dnmt1 affects the methylation modification and gene expression changes during mouse oocyte vitrification. Theriogenology.

[123] Chen H., Zhang L., Deng T., Zou P., Wang Y., Quan F., Zhang Y. (2016). Effects of oocyte vitrification on epigenetic status in early bovine embryos. Theriogenology.

[124] Chen H., Zhang L., Wang Z., Chang H., Xie X., Fu L., Zhang Y., Quan F. (2019). Resveratrol improved the developmental potential of oocytes after vitrification by modifying the epigenetics. Mol. Reprod. Dev..

[125] Wang Z., Xu L., He F. (2010). Embryo vitrification affects the methylation of the H19/Igf2 differentially methylated domain and the expression of H19 and Igf2. Fertil. Steril..

[126] Zhao Y.-H., Wang J.-J., Zhang P.-P., Hao H.-S., Pang Y.-W., Wang H.-Y., Du W.-H., Zhao S.-J., Ruan W.-M., Zou H.-Y. (2020). Oocyte IVM or vitrification significantly impairs DNA methylation patterns in blastocysts as analysed by single-cell whole-genome methylation sequencing. Reprod. Fertil. Dev..

[127] Ying L., Xiang-Wei F., Jun-Jie L., Dian-Shuai Y., Shi-En Z. (2014). DNA methylation pattern in mouse oocytes and their in vitro fertilized early embryos: Effect of oocyte vitrification. Zygote.

[128] De Munck N., Petrussa L., Verheyen G., Staessen C., Vandeskelde Y., Sterckx J., Bocken G., Jacobs K., Stoop D., De Rycke M. (2015). Chromosomal meiotic segregation, embryonic developmental kinetics and DNA (hydroxy) methylation analysis consolidate the safety of human oocyte vitrification. Basic Sci. Reprod. Med..

[129] Liu M.-H., Zhou W.-H., Chu D.-P., Fu L., Sha W., Li Y. (2017). Ultrastructural Changes and Methylation of Human Oocytes Vitrified at the Germinal Vesicle Stage and Matured in vitro after Thawing. Gynecol. Obstet. Investig..

[130] Al-Khtib M., Perret A., Khoueiry R., Ibala-Romdhane S., Blachère T., Greze C., Lornage J., Lefèvre A. (2011). Vitrification at the germinal vesicle stage does not affect the methylation profile of H19 and KCNQ1OT1 imprinting centers in human oocytes subsequently matured in vitro. Fertil. Steril..

[131] Cantatore C., George J.S., Depalo R., D’amato G., Moravek M., Smith G.D. (2021). Mouse oocyte vitrification with and without dimethyl sulfoxide: Influence on cryo-survival, development, and maternal imprinted gene expression. J. Assist. Reprod. Genet..

[132] Jahangiri M., Shahhoseini M., Movaghar B. (2014). H19 and MEST gene expression and histone modification in blastocysts cultured from vitrified and fresh two-cell mouse embryos. Reprod. Biomed. Online.

[133] Movahed E., Shabani R., Hosseini S., Shahidi S., Salehi M. (2020). Interfering effects of in vitro fertilization and vitrification on expression of Gtl2 and Dlk1 in mouse blastocysts. Int. J. Fertil. Steril..

[134] Barberet J., Romain G., Binquet C., Guilleman M., Bruno C., Ginod P., Chapusot C., Choux C., Fauque P. (2021). Do frozen embryo transfers modify the epigenetic control of imprinted genes and transposable elements in newborns compared with fresh embryo transfers and natural conceptions?. Fertil. Steril..

[135] Yao J.-F., Huang Y.-F., Huang R.-F., Lin S.-X., Guo C.-Q., Hua C.-Z., Wu P.-Y., Hu J.-F., Li Y.-Z. (2020). Effects of Vitrification on the Imprinted Gene Snrpn in Neonatal Placental Tissue. Reprod. Dev. Med..

[136] Huo Y., Yuan P., Qin Q., Yan Z., Yan L., Liu P., Li R., Yan J., Qiao J. (2020). Effects of vitrification and cryostorage duration on single-cell RNA-Seq profiling of vitrified-thawed human metaphase II oocytes. Front. Med..

[137] Suo L., Meng Q., Pei Y., Fu X., Wang Y., Bunch T.D., Zhu S. (2010). Effect of cryopreservation on acetylation patterns of lysine 12 of histone H4 (acH4K12) in mouse oocytes and zygotes. J. Assist. Reprod. Genet..

[138] Jahangiri M., Shahhoseini M., Movaghar B. (2018). The Effect of Vitrification on Expression and Histone Marks of Igf2 and Oct4 in Blastocysts Cultured from Two-Cell Mouse Embryos. Cell J..

[139] Chatterjee A., Saha D., Niemann H., Gryshkov O., Glasmacher B., Hofmann N. (2016). Effects of cryopreservation on the epigenetic profile of cells. Cryobiology.

[140] Estudillo E., Jiménez A., Bustamante-Nieves P.E., Palacios-Reyes C., Velasco I., López-Ornelas A. (2021). Cryopreservation of Gametes and Embryos and Their Molecular Changes. Int. J. Mol. Sci..

[141] Yan L.-Y., Yan J., Qiao J., Zhao P.-L., Liu P. (2010). Effects of oocyte vitrification on histone modifications. Reprod. Fertil. Dev..

[142] Maldonado M.B.C., Penteado J.C.T., Faccio B.M.C., Lopes F.L., Arnold D.R. (2015). Changes in tri-methylation profile of lysines 4 and 27 of histone H3 in bovine blastocysts after cryopreservation. Cryobiology.

[143] Stigliani S., Moretti S., Anserini P., Casciano I., Venturini P.L., Scaruffi P. (2015). Storage time does not modify the gene expression profile of cryopreserved human metaphase II oocytes. Hum. Reprod..

[144] Yan Y., Zhang Q., Yang L., Zhou W., Ni T., Yan J. (2022). Pregnancy and neonatal outcomes after long-term vitrification of blastocysts among 6900 patients after their last live birth. Fertil. Steril..

[145] Bouillon C., Leandri R., Desch L., Ernst A., Bruno C., Cerf C., Chiron A., Souchay C., Burguet A., Jimenez C. (2016). Does Embryo Culture Medium Influence the Health and Development of Children Born after In Vitro Fertilization?. PLoS ONE.

[146] Sciorio R., Tramontano L., Rapalini E., Bellaminutti S., Bulletti F.M., D’Amato A., Manna C., Palagiano A., Bulletti C., Esteves S.C. (2022). Risk of genetic and epigenetic alteration in children conceived following ART: Is it time to return to nature whenever possible?. Clin. Genet..

[147] Sciorio R., El Hajj N. (2022). Epigenetic Risks of Medically Assisted Reproduction. J. Clin. Med..

[148] Barberet J., Barry F., Choux C., Guilleman M., Karoui S., Simonot R., Bruno C., Fauque P. (2020). What impact does oocyte vitrification have on epigenetics and gene expression?. Clin. Epigenet..

[149] Hezavehei M., Sharafi M., Kouchesfahani H.M., Henkel R., Agarwal A., Esmaeili V., Shahverdi A. (2018). Sperm cryopreservation: A review on current molecular cryobiology and advanced approaches. Reprod. Biomed. Online.

[150] Tran K.T.D., Valli-Pulaski H., Colvin A., E Orwig K. (2022). Male fertility preservation and restoration strategies for patients undergoing gonadotoxic therapies. Biol. Reprod..

[151] Bunge R.G., Sherman J.K. (1953). Fertilizing Capacity of Frozen Human Spermatozoa. Nature.

[152] Riva N.S., Ruhlmann C., Iaizzo R.S., Marcial Lopez C.A., Martinez A.G. (2018). Comparative analysis between slow freezing and ultra-rapid freezing for human sperm cryopreservation. JBRA Assist. Reprod..

[153] Isachenko V., Maettner R., Petrunkina A.M., Sterzik K., Mallmann P., Rahimi G., Sanchez R., Risopatron J., Damjanoski I., Isachenko E. (2012). Vitrification of human ICSI/IVF spermatozoa without cryoprotectants: New capillary technology. J. Androl..

[154] Aitken R., De Iuliis G. (2009). On the possible origins of DNA damage in human spermatozoa. Mol. Hum. Reprod..

[155] Wu Q., Ni X. (2015). ROS-mediated DNA methylation pattern alterations in carcinogenesis. Curr. Drug Targets.

[156] Ziech D., Franco R., Pappa A., Panayiotidis M.I. (2011). Reactive Oxygen Species (ROS)––Induced genetic and epigenetic alterations in human carcinogenesis. Mutat. Res. Mol. Mech. Mutagen..

[157] Valipour J., Nashtaei M.S., Khosravizadeh Z., Mahdavinezhad F., Nekoonam S., Esfandyari S., Amidi F. (2020). Effect of sulforaphane on apoptosis, reactive oxygen species and lipids peroxidation of human sperm during cryopreservation. Cryobiology.

[158] Kläver R., Tüttelmann F., Bleiziffer A., Haaf T., Kliesch S., Gromoll J. (2013). DNA methylation in spermatozoa as a prospective marker in andrology. Andrology.

[159] El Hajj N., Zechner U., Schneider E., Tresch A., Gromoll J., Hahn T., Schorsch M., Haaf T. (2011). Methylation Status of Imprinted Genes and Repetitive Elements in Sperm DNA from Infertile Males. Sex. Dev..

[160] Marques C.J., Francisco T., Sousa S., Carvalho F., Barros A., Sousa M. (2010). Methylation defects of imprinted genes in human testicular spermatozoa. Fertil. Steril..

[161] Poplinski A., Tüttelmann F., Kanber D., Horsthemke B., Gromoll J. (2010). Idiopathic male infertility is strongly associated with aberrant methylation of MEST and IGF2/H19 ICR1. Int. J. Androl..

[162] Laurentino S., Beygo J., Nordhoff V., Kliesch S., Wistuba J., Borgmann J., Buiting K., Horsthemke B., Gromoll J. (2014). Epigenetic germline mosaicism in infertile men. Hum. Mol. Genet..

[163] de Mello F., Garcia J.S., Godoy L.C., Depincé A., Labbé C., Streit D.P. (2017). The effect of cryoprotectant agents on DNA methylation patterns and progeny development in the spermatozoa of Colossoma macropomum. Gen. Comp. Endocrinol..

[164] Depincé A., Gabory A., Dziewulska K., Le Bail P., Jammes H., Labbé C. (2019). DNA methylation stability in fish spermatozoa upon external constraint: Impact of fish hormonal stimulation and sperm cryopreservation. Mol. Reprod. Dev..

[165] Salehi M., Mahdavi A.H., Sharafi M., Shahverdi A. (2020). Cryopreservation of rooster semen: Evidence for the epigenetic modifications of thawed sperm. Theriogenology.

[166] He W., Sun Υ., Zhang S., Feng X., Xu M., Dai J., Ni X., Wang X., Wu Q. (2020). Profiling the DNA methylation patterns of imprinted genes in abnormal semen samples by next-generation bisulfite sequencing. J. Assist. Reprod. Genet..

[167] Khosravizadeh Z., Hassanzadeh G., Bazzaz J.T., Alizadeh F., Totonchi M., Salehi E., Khodamoradi K., Khanehzad M., Hosseini S.R., Abolhassani F. (2020). The effect of cryopreservation on DNA methylation patterns of the chromosome 15q11–q13 region in human spermatozoa. Cell Tissue Bank.

[168] Bogle O.A., Kumar K., Attardo-Parrinello C., Lewis S.E.M., Estanyol J.M., Ballescà J.L., Oliva R. (2016). Identification of protein changes in human spermatozoa throughout the cryopreservation process. Andrology.

[169] Santi D., De Vincentis S., Magnani E., Spaggiari G. (2017). Impairment of sperm DNA methylation in male infertility: A meta-analytic study. Andrology.

[170] Güngör İ.H., Tektemur A., Arkali G., Cinkara S.D., Acisu T.C., Koca R.H., Önalan E.E., Kaya Ş.Ö., Kizil M., Sönmez M. (2021). Effect of freeze–thawing process on lipid peroxidation, miRNAs, ion channels, apoptosis and global DNA methylation in ram spermatozoa. Reprod. Fertil. Dev..

